# A Green-Synthesized Zr-Tb Bimetallic MOF: Ratiometric Fluorescent Probe for Selective and Sensitive Detection of Ciprofloxacin

**DOI:** 10.3390/molecules31091423

**Published:** 2026-04-25

**Authors:** Yue Wang, Binbin Lu, Shu Li, Chaofan Ma, Ying Zou, Guoyuan Li, Shuo Liu

**Affiliations:** 1 School of Energy and Chemical Engineering, Tianjin Renai College, Tianjin 301636, China; wangyue102@tju.edu.cn (Y.W.); 15692201260@163.com (B.L.); 15735524939@163.com (C.M.); 13920039337@163.com (Y.Z.); 13609340415@163.com (G.L.); 2 Technology Center of Shenyang Customs, Shenyang 110016, China; linda_0915@126.com

**Keywords:** Zr–Tb bimetallic MOF, green synthesis, ratiometric fluorescence, ciprofloxacin detection

## Abstract

The widespread residual ciprofloxacin (CIP) poses severe environmental and health risks, demanding efficient detection methods. Herein, a Zr–Tb bimetallic MOF (ZTM) was green-synthesized via a room-temperature aqueous route with disodium terephthalate as ligand, and developed as a ratiometric fluorescent probe for CIP detection. Structural characterization confirmed Tb^3+^ was successfully incorporated into the Zr-MOF framework, endowing ZTM with high stability and excellent luminescence. The absorption edge of ZTM (320–330 nm) overlapped with CIP’s 330 nm absorption peak, so 327 nm was selected as the excitation wavelength. Under this excitation, ZTM showed a strong Tb^3+^ emission at 657 nm; upon CIP addition, the 657 nm peak was quenched, while the 491 nm emission was enhanced, realizing a distinct ratiometric response. The ratio I_491_/I_657_ was linear with CIP concentration (0.5–25 μM, R^2^ = 0.992), with a limit of detection far below the statutory 30 μM limit (0.16 μM). ZTM also exhibited excellent selectivity, good pH tolerance (5.0–8.0) and rapid response (1 min). Mechanism analysis revealed that the response was mainly due to the inner filter effect (IFE) between ZTM and CIP. This work provides a green-synthesized MOF probe for sensitive and selective CIP detection in environmental samples.

## 1. Introduction

The overuse and abuse of antibiotics have caused serious environmental pollution and drug resistance problems, which have become a global public health threat [1,2,3]. Ciprofloxacin (CIP), as a typical fluoroquinolone antibiotic, is widely used in clinical treatment, animal husbandry and aquaculture due to its broad antibacterial spectrum and good therapeutic effect. However, it is difficult to be completely metabolized by organisms, resulting in a large number of residues in water, soil and food chains. Long-term exposure to low-concentration antibiotic residues will not only destroy the ecological balance, but also induce the production of drug-resistant bacteria and drug-resistant genes, which will eventually endanger human health [4,5,6]. Therefore, it is particularly important to establish a simple, rapid, sensitive and reliable method for the detection of CIP in environmental samples [7,8,9,10].

Among various detection strategies, fluorescence sensing has garnered considerable attention owing to its high sensitivity, fast response, low cost, simple operation, and potential for on-site visualization [11,12]. Compared with single-emission fluorescence detection, ratiometric fluorescence sensing can provide built-in correction for environmental interferences and instrument fluctuations, resulting in improved accuracy, enhanced anti-interference ability, and lower detection limits [13,14]. Therefore, the development of ratiometric fluorescent probes for efficient and selective detection of CIP is of great practical significance [15,16,17].

Metal–organic frameworks (MOFs), as a class of crystalline porous materials, have been widely utilized in fluorescence sensing on account of their large specific surface area, adjustable pore structure, abundant active sites, and designable optical properties [18,19,20]. Lanthanide-based MOFs possess unique luminescence features including large Stokes shifts, narrow emission bands, and high fluorescence stability, making them ideal candidates for fluorescent sensing [21,22,23,24]. However, single-lanthanide MOFs often suffer from insufficient structural stability and relatively low sensing response. By contrast, bimetallic MOFs that integrate a structurally stable metal ion and a luminescent lanthanide ion can combine the merits of each component, thus synergistically enhancing both framework stability and sensing performance [25,26].

Most reported MOF-based fluorescent probes are prepared via solvothermal methods, which require high temperature, long reaction times, and toxic organic solvents, conflicting with the concept of green chemistry [27]. In our previous work, we have successfully developed a green-synthesized europium-based MOF for ratiometric fluorescence sensing of Cu^2+^ via a room-temperature aqueous approach, which verified the feasibility and superiority of green synthesis strategies for fabricating luminescent MOF-based sensors [28]. Motivated by these favorable findings, we rationally constructed a bimetallic Zr–Tb MOF (ZTM) by introducing Tb^3+^ into a robust Zr-matrix framework using disodium terephthalate (BDC-Na_2_) as the organic ligand. This facile room-temperature aqueous synthesis was designed to clarify the synergistic effect of dual-metal sites on fluorescence sensing toward antibiotic residues. The resulting ZTM exhibited an excellent ratiometric response toward CIP: the emerging emission around 491 nm was significantly enhanced, while the intrinsic characteristic emission of Tb^3+^ at approximately 657 nm was remarkably quenched. This probe shows high selectivity, excellent sensitivity, and favorable stability against pH, temperature, and interfering substances. This work not only provides a green and scalable strategy for constructing disodium terephthalate-coordinated bimetallic lanthanide–Zr MOFs but also expands their application in the rapid fluorescence sensing of antibiotic residues, and lays a solid foundation for the subsequent exploration of dual-lanthanide MOF systems for multi-target sensing.

## 2. Results and Discussion

### 2.1. Structural and Morphological Characterization of ZTM, ZM and TM

To realize the green synthesis of a lanthanide-based MOF via a facile room-temperature aqueous route, disodium terephthalate (BDC-Na2) was selected as the organic ligand (instead of terephthalic acid, BDC) to construct the Zr–Tb bimetallic MOF (ZTM) [29,30]. For comparative study, the monometallic Zr-MOF (ZM) and Tb-MOF (TM) were also synthesized under the same experimental conditions.

**Figure 1:**
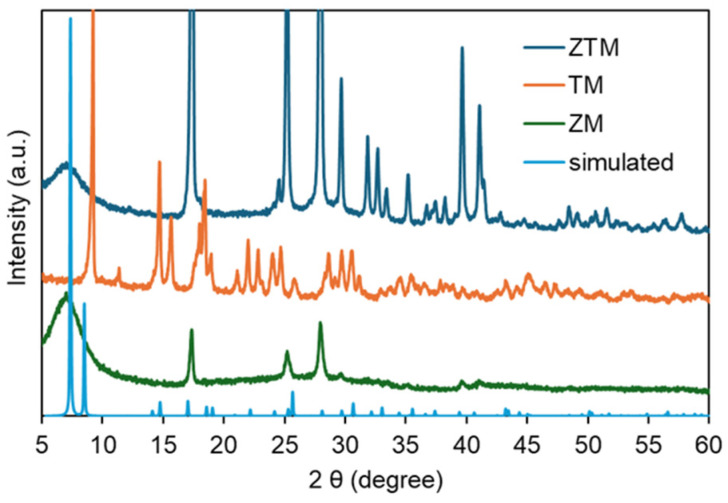
XRD patterns of ZTM, ZM, TM and simulated UiO-66.

X-ray diffraction (XRD) analysis was conducted to identify the crystalline structures and phase purities of ZM, TM and ZTM, with the simulated pattern of the parent Zr-MOF framework (UiO-66) as a reference. As shown in Figure 1, the simulated pattern (light blue) displayed sharp and well-defined characteristic diffraction peaks, representing the ideal crystalline structure of the Zr-MOF. ZM (green) exhibited diffraction peaks that matched well with the simulated pattern at key 2θ positions (e.g., ~5.5°, ~17°, ~26°, ~29°), confirming the successful formation of the Zr-MOF framework with high crystallinity [31,32]. In contrast, TM (orange) showed a completely different set of diffraction peaks, with multiple sharp peaks distributed across the 5–60° range, indicating a distinct crystalline structure formed by the coordination of Tb^3+^ with BDC-Na2 [33]. Notably, ZTM (blue) not only retained all the main characteristic peaks of ZM that aligned with the simulated pattern, but also introduced several additional weak peaks that coincided with the characteristic peaks of TM. This result confirmed that Tb^3+^ was successfully incorporated into the Zr-MOF framework through co-coordination with BDC-Na2, forming a bimetallic crystalline structure that inherited the main framework of ZM while integrating the structural features of TM. The well-preserved main peaks of ZM in ZTM demonstrated that the introduction of Tb^3+^ did not destroy the original Zr-MOF skeleton, and the appearance of TM-derived weak peaks further verified the successful coexistence of Zr^4+^ and Tb^3+^ in the bimetallic framework, rather than a simple physical mixture of ZM and TM.

**Figure 2:**
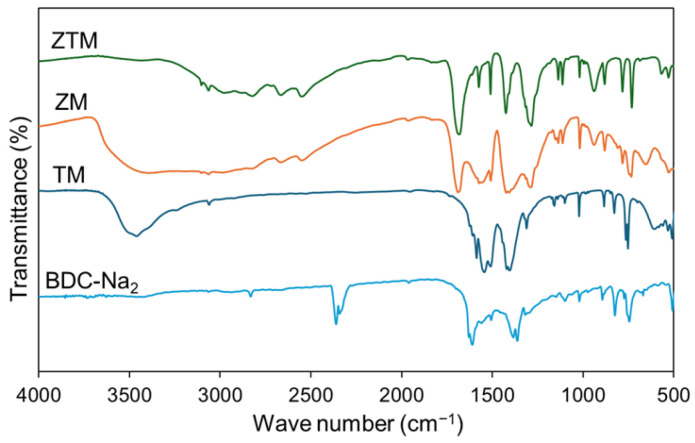
FT-IR results of ZTM, ZM, TM and BDC-Na2.

Fourier transform infrared (FT-IR) spectroscopy was used to investigate the functional group changes and verify the metal–ligand coordination interactions in ZM, TM and ZTM, with BDC-Na2 and BDC as the control. As shown in Figure 2, the characteristic absorption peaks of carboxylate anions appeared at ~1560 cm^−1^ and ~1410 cm^−1^ for BDC-Na2, corresponding to the asymmetric and symmetric stretching vibrations of –COO^−^, respectively, and no absorption peak was detected at ~1700 cm^−1^ (BDC, Figure S1), the typical characteristic peak of free –COOH groups, which was consistent with the structural characteristics of carboxylate salts. After the formation of MOFs, ZM showed a characteristic absorption peak at ~700 cm^−1^, assigned to the stretching vibration of Zr–O bonds, confirming the successful construction of the Zr-MOF framework [30,31,34]. TM exhibited a distinct absorption peak at ~600 cm^−1^, corresponding to the stretching vibration of Tb–O bonds, which was direct evidence for the coordination between Tb^3+^ and carboxylate ligands. For ZTM, the FT-IR spectrum retained the characteristic Zr–O stretching peak at ~700 cm^−1^ and also showed a weak but discernible Tb–O stretching peak at ~600 cm^−1^, with the coordination carboxylate peaks consistent with those of ZM in the main trend. In addition, all three MOFs retained the weak absorption peaks of aromatic C–H stretching vibration at ~3000 cm^−1^ and benzene ring skeleton vibration at ~1600 cm^−1^, indicating that the aromatic ring skeleton of BDC-Na2 was not destroyed during the coordination reaction. These FT-IR results further confirmed the successful formation of ZTM as a bimetallic MOF via the co-coordination of Zr^4+^ and Tb^3+^ with BDC-Na2.

**Figure 3:**
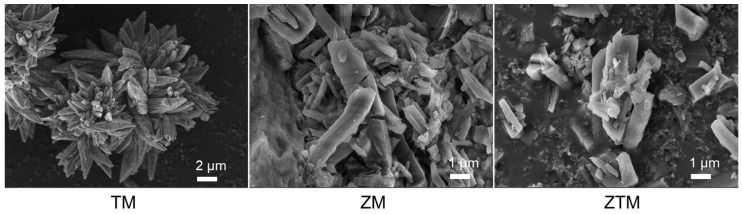
SEM results of TM, ZM and ZTM.

Scanning electron microscopy (SEM) was employed to observe the micro-morphology of ZM, TM and ZTM. As shown in Figure 3, ZM displayed a regular crystalline morphology, consistent with its high crystallinity revealed by XRD. TM showed an irregular, agglomerated morphology with no obvious crystalline shape, reflecting its distinct structural characteristics. ZTM inherited the regular crystalline morphology of ZM, with uniform particle distribution and no severe agglomeration, indicating that the introduction of Tb^3+^ did not disrupt the basic framework structure of Zr-MOF. The well-dispersed morphology of ZTM is beneficial for exposing active sites and adsorbing target analytes, laying a foundation for its subsequent sensing performance. Additionally, energy dispersive spectroscopy (EDS) mapping results confirmed the homogeneous distribution of Zr, Tb and O elements throughout the ZTM sample (Figure S2). The actual molar ratio of Zr to Tb was approximately 0.62:1. Notably, there are almost no Na and N elements, which directly confirms that residual Na^+^ from the BDC-Na2 and NO_3_^−^ from the metal nitrate precursors were completely removed during the washing process, verifying the high purity of the as-synthesized ZTM and the complete coordination reaction between the metal centers and the organic ligand.

**Figure 4:**
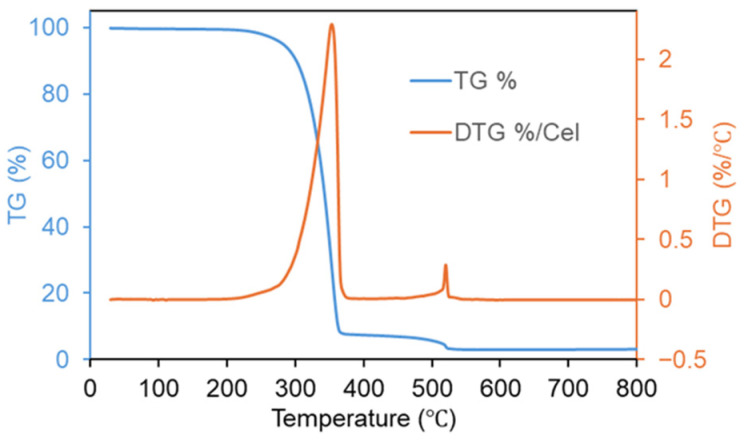
TGA curve of ZTM.

Thermogravimetric analysis (TGA) was carried out under air atmosphere to evaluate the thermal stability of ZTM, a crucial physicochemical property for its practical fluorescence sensing application. As shown in Figure 4, the TGA curve of ZTM exhibited three distinct weight loss stages, which was consistent with the typical thermal decomposition behavior of MOF materials. The first weight loss stage occurred below 150 °C, with a small weight loss ratio, which was attributed to the evaporation of physically adsorbed water on the surface and in the pore channels of ZTM. The second stage (150–500 °C) was the main thermal decomposition process, with the fastest weight loss rate observed around 300–400 °C, corresponding to the gradual cleavage of M–O coordination bonds and the subsequent decomposition of the organic ligand disodium terephthalate coordinated with Zr^4+^ and Tb^3+^. ZTM remained thermally stable up to approximately 250 °C without significant weight loss, demonstrating good thermal resistance for environmental sensing applications.

**Figure 5:**
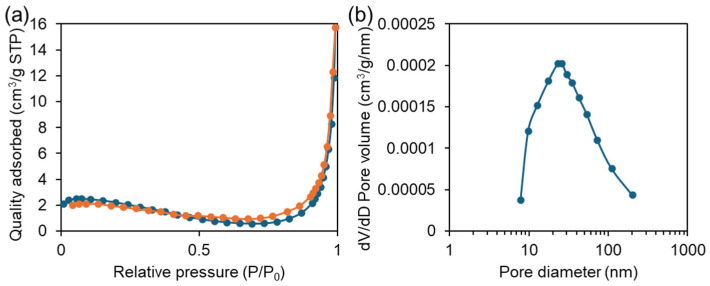
(**a**) N_2_ adsorption–desorption isotherm and (**b**) pore size distribution of ZTM.

N_2_ adsorption–desorption (BET) measurements were performed to determine the porous structure parameters of ZTM. As shown in Figure 5a, the N_2_ adsorption–desorption isotherm of ZTM was a typical Type IV isotherm with a distinct H3 hysteresis loop, a characteristic feature of mesoporous materials, indicating that ZTM possesses a developed mesoporous structure. The specific surface area of ZTM was calculated to be 10.61 m^2^·g^−1^, with a total pore volume of 0.024 cm^3^·g^−1^ and an average pore size of 9.17 nm. From the pore size distribution in Figure 5b, the pore structure of ZTM is dominated by mesopores (approximately 10–100 nm). The mesoporous structure of ZTM can facilitate the diffusion of CIP molecules into the framework, which is beneficial for the interaction between the probe and the target analyte.

### 2.2. Optical Properties of ZTM, ZM and TM

To explore the feasibility of ZTM as a fluorescent probe for ciprofloxacin (CIP) detection, its optical properties were systematically investigated via UV-Vis absorption and fluorescence spectroscopy, with the absorption spectrum of CIP as a reference.

**Figure 6:**
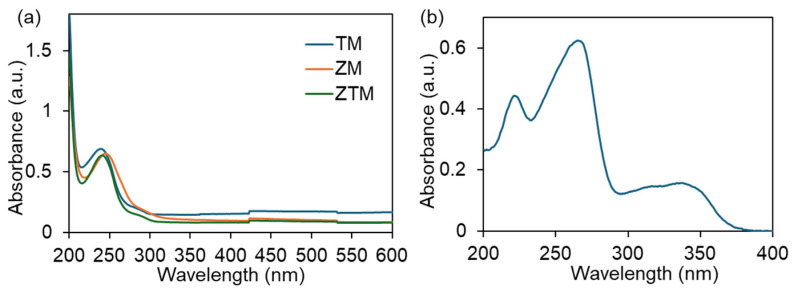
(**a**) UV–vis spectra of TM, ZM and ZTM. (**b**) UV–vis spectra of CIP.

The UV-Vis absorption spectra of ZM, TM and ZTM were measured in the range of 200–650 nm, as shown in Figure 6a. All three materials exhibited a similar absorption profile, with a prominent characteristic absorption peak in the ultraviolet region, which is attributed to the π→π* electronic transition of the aromatic benzene ring in the coordinated BDC-Na_2_ ligand. Specifically, TM (blue curve) showed a maximum absorption peak at ~239 nm, ZM (orange curve) at ~241 nm, and ZTM (yellow curve) at ~244 nm. The gradual red-shift in the maximum absorption wavelength from TM to ZM to ZTM further verified the distinct coordination environments of Tb^3+^ and Zr^4+^ with the BDC-Na_2_ ligand, confirming the successful co-doping of Tb^3+^ into the Zr-MOF framework to form the bimetallic ZTM structure. The absorption edge of ZTM was located in the range of 320–330 nm, where the material still maintained a significant absorbance.

The UV-Vis absorption spectrum of CIP, as shown in Figure 6b, exhibited three distinct characteristic absorption peaks in the ultraviolet region—a weak secondary peak at ~225 nm, a strong maximum absorption peak at ~270 nm, and a broad, weak absorption peak centered at ~330 nm—with a distinct absorption tail extending to ~380 nm. These peaks are assigned to the π→π* transition of the aromatic quinoline ring (225 nm and 270 nm) and the n→π* transition of the carbonyl group in the fluoroquinolone structure (330 nm), which are typical absorption features of ciprofloxacin. Notably, the absorption edge of ZTM (320–330 nm) overlaps well with the characteristic absorption peak of CIP at ~330 nm. Therefore, 327 nm was chosen as the excitation wavelength to investigate the fluorescence response of ZTM toward CIP.

**Figure 7:**
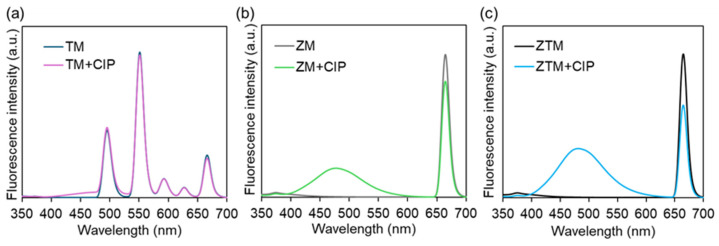
The fluorescence curves of TM (**a**), ZM (**b**), ZTM (**c**) with or without CIP (25 μM).

The fluorescence emission spectra of ZTM, ZM and TM before and after the addition of CIP were then tested under the excitation wavelength of 327 nm. As shown in Figure 7a, TM (dark blue curve) displayed multiple distinct characteristic emission peaks of Tb^3+^ at ~490 nm, ~545 nm, ~585 nm and ~655 nm, which are assigned to the typical ^5^D_4_→^7^Fⱼ (J = 6, 5, 4, 3) f-f transitions of Tb^3+^ [35]. After the introduction of CIP (pink curve), the emission intensities of all Tb^3+^ characteristic peaks showed only a marginal increase, with no significant change in the overall fluorescence profile, demonstrating that TM lacks an effective sensing response to CIP. As shown in Figure 7b, ZM (gray curve) exhibited a strong characteristic emission peak centered at ~657 nm. This peak in ZM originates from ligand-centered fluorescence rather than Tb^3+^ emission, since ZM contains no Tb element. Upon the addition of CIP (green curve), only a slight decrease in the 657 nm emission intensity was observed, accompanied by an enhancement of the broad emission band in the 450–550 nm range. Interestingly, upon addition of CIP, the emission peak at 657 nm decreased, while the peak at and ~491 nm increased more significantly compared with ZM (Figure 7c). This distinct dual-signal response is a typical feature of ratiometric fluorescence sensing, which can effectively eliminate the interference of environmental factors and instrument fluctuations, thus improving the accuracy and reliability of detection.

### 2.3. Fluorescence Sensing Performance of ZTM Toward CIP

**Figure 8:**
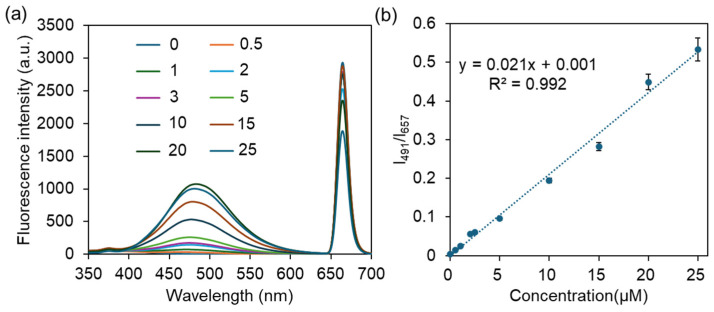
(**a**) The fluorescence spectra of ZTM with increasing CIP. (**b**) The linear relationship between the fluorescence ratio I_491_/I_657_ and the concentration of CIP.

To assess the sensitivity of ZTM for CIP detection, the fluorescence emission spectra of ZTM with varying concentrations of CIP (0–25 μM) were measured at an excitation wavelength of 327 nm. As shown in Figure 8a, with the gradual increase in CIP concentration, the peak at 657 nm was gradually quenched, while the broad emission band centered at ~491 nm was continuously enhanced, exhibiting a clear and reversible ratiometric fluorescence change. The fluorescence intensity ratio (I_491_/I_657_) was selected as the analytical signal for quantitative detection, which exhibited an excellent linear relationship with CIP concentration in the range of 0.5–25 μM, with a high correlation coefficient (R^2^ = 0.992, Figure 8b). The limit of detection (LOD) was calculated to be 0.16 μM according to the 3σ/S rule, which is significantly lower than the maximum residue limit of 30 μM (100 ng/mL) for CIP stipulated by the Ministry of Agriculture of China and the European Commission in practical samples, demonstrating the high sensitivity of ZTM for trace CIP detection [36]. Moreover, compared with the reported MOF-based fluorescent detection of CIP, the linear ranges and LOD were similar to or better than those reported in the literature [37] (0–24.3 μM, 0.27 μM), [38] (0–75 μM, 1.67 μM), [39] (3–20 μM, 0.315 μM) and [40] (0–22.2 μM, 1.07 μM). To further verify the superiority of the bimetallic ZTM probe, the fluorescence response of monometallic ZM toward CIP was investigated under identical conditions for comparison (Figure S3). The fluorescence intensity ratio I_479_/I_654_ presented a narrow linear relationship with a relatively low correlation coefficient (2–20 μM, R^2^ = 0.9834, Figure S3b), demonstrating the unique advantage of the bimetallic ZTM system over the monometallic ZM.

Combined with the UV–vis spectral characteristics and fluorescence response behaviors, the ratiometric sensing mechanism of ZTM toward CIP was dominated by the inner filter effect (IFE) [7,19]. As confirmed in Figure 6, the absorption edge of ZTM (320–330 nm) exhibits effective spectral overlap with the characteristic absorption peak of CIP at ~330 nm. When excited at 327 nm, CIP competitively absorbs the incident excitation light energy that should be captured by ZTM, which reduces the ligand-to-metal energy transfer efficiency in the bimetallic framework and thus remarkably quenches the intrinsic characteristic emission of Tb^3+^ at 657 nm [6,20]. Meanwhile, the enhanced broad emission at ~491 nm originates from the intrinsic emission of CIP itself and the recovered ligand-centered emission of ZTM, forming a distinct ratiometric fluorescence response [19,22]. Furthermore, specific non-covalent interactions including hydrogen bonding and π–π stacking between ZTM and CIP may also contribute to the regulated fluorescence output. These interactions help stabilize the excited state of the probe and suppress non-radiative relaxation pathways. Meanwhile, the high specific surface area and porous structure of ZTM provide abundant accessible recognition sites toward CIP.

**Figure 9:**
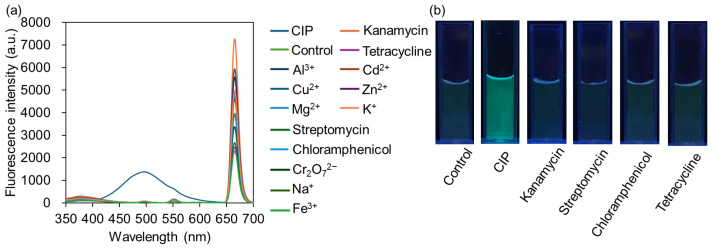
(**a**) ZTM fluorescence spectrum before and after reaction with different kinds of metal ions and antibiotics (25 µM). (**b**) Photographs of ZTM with different kinds of antibiotics (25 µM) under UV light (302 nm).

Selectivity is a critical indicator for evaluating the practical application potential of fluorescent probes. To investigate the selectivity of ZTM toward CIP, the fluorescence responses of ZTM to various interfering substances, including other common antibiotics (kanamycin, streptomycin, tetracycline, chloramphenicol, ofloxacin, norfloxacin) and common coexisting ions in environmental water (Na^+^, K^+^, Cd^2+^, Mg^2+^, Cu^2+^, Al^3+^, Zn^2+^, Fe^3+^, Cr_2_O_7_^2−^), were measured under identical conditions. As shown in Figure 9a, only the addition of CIP induced a distinct ratiometric fluorescence response; the emission peak at 491 nm was significantly enhanced, while the peak at 657 nm was remarkably quenched. In contrast, none of the other interfering substances triggered the emergence of the 491 nm emission peak—even if some fluctuations in the 657 nm peak intensity were observed, the characteristic dual-signal ratiometric response of ZTM was not generated. The visual selectivity was further verified by the photographs under UV light (302 nm). As shown in Figure 9b, the ZTM dispersion with CIP showed a distinct fluorescence color change compared to the control group and other interfering substance groups, which maintained negligible fluorescence variation. These results from both spectral analysis and visual observation confirm that ZTM shows good selectivity toward many common antibiotics and metal ions in environmental water matrices. It should be noted that this study mainly focuses on the feasibility of green-synthesized ratiometric fluorescence sensing for CIP. The specific recognition toward ciprofloxacin over its structurally similar fluoroquinolone analogs will be further explored in our future work to develop a more specific sensing platform.

**Figure 10:**
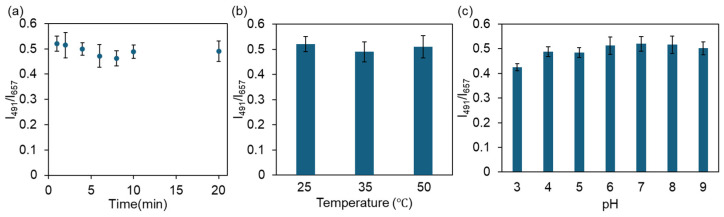
Effects of response time (**a**), temperature (**b**) and pH (**c**) on the detection of CIP.

To further evaluate the practical application potential of ZTM, the influences of reaction time, temperature and solution pH on the ratiometric fluorescence sensing performance toward CIP were investigated. As shown in Figure 10a, the fluorescence intensity ratio I_491_/I_657_ reached a stable equilibrium within 1 min after CIP addition and remained constant thereafter, demonstrating rapid sensing kinetics that enables efficient on-site detection of trace CIP residues. Figure 10b shows that the I_491_/I_657_ ratio exhibited no obvious fluctuation in the temperature range of 25–50 °C, indicating the excellent thermal stability of ZTM during the detection process. As illustrated in Figure 10c, the I_491_/I_657_ ratio remained stable in the pH range of 5.0–8.0, revealing good pH tolerance of ZTM and its adaptability for CIP detection in natural water samples under neutral or weakly acidic/alkaline conditions.

## 3. Materials and Methods

### 3.1. Reagents and Chemicals

Terbium nitrate hexahydrate (Tb(NO_3_)_3_·6H_2_O), zirconium chloride (ZrCl_4_), disodium terephthalate (BDC-Na2), sodium chloride, potassium chloride, aluminum chloride, magnesium chloride, copper chloride, calcium chloride, zinc chloride, and potassium dichromate were of analytical grade and bought from Macklin Biochemical Co., Ltd. (Shanghai, China). The water used in this work was ultrapure water.

### 3.2. Synthesis of ZM, TM and ZTM

For ZTM, 45 mg terbium nitrate hexahydrate and 23 mg zirconium chloride were dissolved in 10 mL of deionized water to obtain solution A. Meanwhile, 21 mg disodium terephthalate was dissolved in another 10 mL of deionized water, denoted as solution B. Subsequently, solution A was slowly added dropwise into solution B under continuous magnetic stirring. After the completion of dropping, the mixed system was further stirred for 30 min to ensure sufficient reaction. The resulting white precipitate was collected by centrifugation at 10,000 rpm for 2 min, washed twice with deionized water, and centrifuged again for subsequent collection. The obtained samples were dispersed in water and stored at 4 °C for further experiments. The final solid products were obtained by vacuum drying (60 °C, 12 h).

ZM was prepared under the identical conditions as ZTM, except that only 46 mg zirconium chloride was used without the addition of terbium nitrate hexahydrate. The total metal ion molar concentration was kept consistent with that of ZTM.

TM was prepared under identical conditions as ZTM, except that only 90 mg terbium nitrate hexahydrate was employed in the absence of zirconium chloride. The total metal ion molar concentration was also kept consistent with that of ZTM.

### 3.3. Characterization

Morphological features were characterized using a scanning electron microscope (SEM, ZEISS Sigma 360, Oberkochen, Germany). Fourier transform infrared spectroscopy (FT-IR, ThermoFisher Scientific, Nicolet iS50, Waltham, MA, USA) was employed to identify surface functional groups using the KBr pellet method in the wavenumber range of 4000–400 cm^−1^. X-ray diffraction (XRD, Rigaku SmartLab, Tokyo, Japan) was utilized to analyze the crystalline structure of the samples. Fluorescence spectra were recorded on an F97 fluorescence spectrophotometer (Shanghai Lengguang Technology, Shanghai, China). The pore structure and specific surface area were determined using a surface area and porosity analyzer (BET, Micromeritics ASAP 2460, Norcross, USA). Thermogravimetric analysis (TGA) was carried out using a thermal analyzer (HITACHI STA200, Tokyo, Japan) in air atmosphere with a heating rate of 10 °C·min^−1^ from room temperature to 800 °C.

### 3.4. Fluorescent Detection of CIP

The aqueous solution of ZTM (0.2 mg/mL) was prepared by dispersing ZTM into ultrapure water under ultrasound for 10 min. Then, 1 mL CIP solution at various concentrations was added to 1 mL of ZTM solution. After incubation for 1 min, fluorescence emission spectra were recorded. All experiments were performed at room temperature and repeated at least three times for reliability.

## 4. Conclusions

In this work, a dual-metal Zr/Tb MOF (ZTM) was successfully synthesized and utilized as a high-performance ratiometric fluorescent probe for sensitive detection of ciprofloxacin. UV-vis results confirmed characteristic absorption differences among TM, ZM and ZTM at 239 nm, 241 nm and 244 nm, verifying the successful construction of bimetallic coordination structure. Under excitation at 327 nm, ZTM exhibited strong characteristic emission of Tb^3+^ at 657 nm, which was remarkably quenched upon addition of CIP, accompanied by obvious enhancement around 491 nm; by contrast, single TM and ZM showed negligible responses, demonstrating the superior sensing capability of ZTM. The as-prepared probe displayed high sensitivity with a low LOD far below the standard limit of 30 μM regulated by the Ministry of Agriculture of China and the European Commission, satisfactory anti-interference ability, suitable pH stability and rapid response (1 min). This study provides a promising luminescent MOF platform for the rapid and accurate monitoring of CIP residues in water.

## Data Availability

Data may be available from the corresponding author, Shuo Liu, upon request.

## Supplementary Materials

The following supporting information can be downloaded at https://www.mdpi.com/article/10.3390/molecules31091423/s1, Figure S1: FT-IR spectra of BDC. Figure S2: EDS mapping images of ZTM. Figure S3: (a) The fluorescence spectra of ZM with increasing CIP. (b) The linear relationship between the fluorescence ratio I479/I654 and the concentration of CIP.
