# Supplementary material for: A Green-Synthesized Zr-Tb Bimetallic MOF: Ratiometric Fluorescent Probe for Selective and Sensitive Detection of Ciprofloxacin
Source: Molecules. 2026 Apr 25;31(9):1423. doi: 10.3390/molecules31091423 (PMC13164965; doi:10.3390/molecules31091423)
Supplement: Supplementary file 1 [file molecules-31-01423-s001.zip › molecules-4261785-supplementary.pdf]

A Green-Synthesized Zr-Tb Bimetallic MOF: Ratiometric Fluorescent Probe for Selective and Sensitive Detection of Ciprofloxacin

Yue Wang [1], Binbin Lu [1], Shu Li [2], Chaofan Ma [1], Ying Zou [1], Guoyuan Li [1] and Shuo Liu [1,*]

[1] School of Energy and Chemical Engineering, Tianjin Renai College, Tianjin 301636, China
[2] Technology Center of Shenyang Customs, Shenyang, 110016, China; linda_0915@126.com
* Correspondence: liushuo@nankai.edu.cn

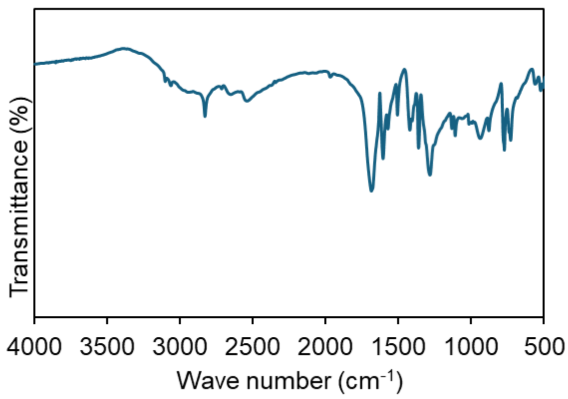

Fig. S1 FT-IR spectra of BDC.

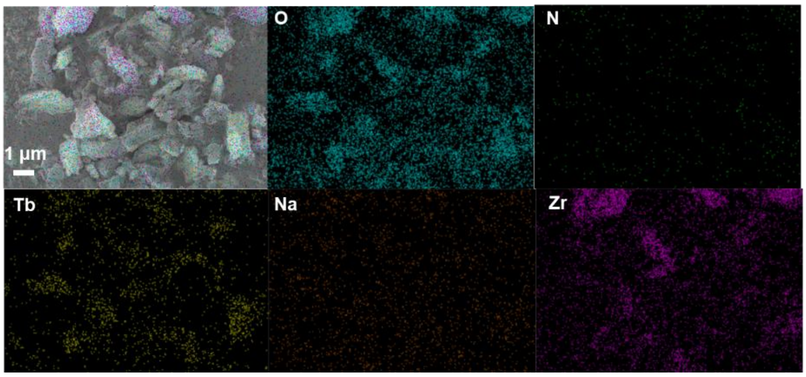

Fig. S2 EDS mapping images of ZTM.

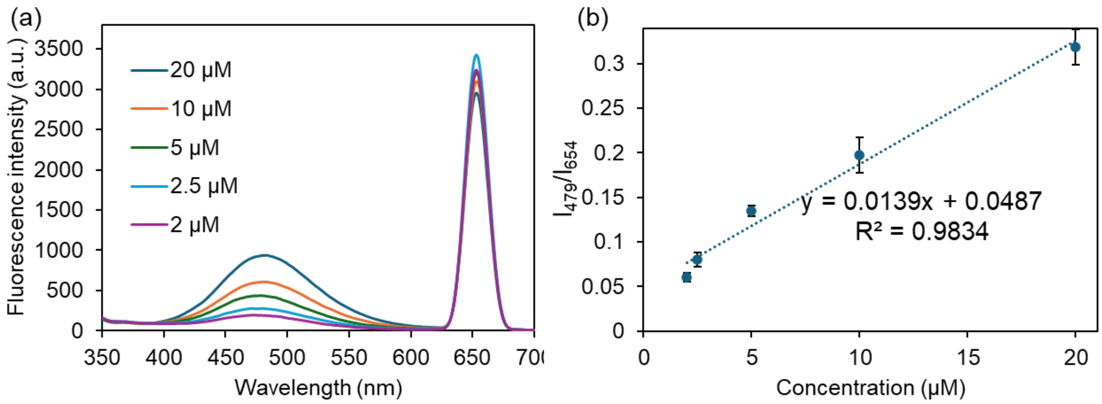

Fig. S3 (a) The fluorescence spectra of ZM with increasing CIP. (b) The linear relationship between the fluorescence ratio I_{479}/I_{654} and the concentration of CIP.
